# Data-driven analysis of anomalous transport and three-wave-coupling effects in an $E \times B$ plasma

**DOI:** 10.1007/s44205-025-00158-6

**Published:** 2025-09-03

**Authors:** Borja Bayón-Buján, Enrique Bello-Benítez, Jiewei Zhou, Mario Merino

**Affiliations:** Department of Aerospace Engineering, Universidad Carlos III de Madrid, Leganés, Spain

## Abstract

The collisionless cross-field electron transport in an $E\times B$ plasma configuration, representative of a Hall thruster, is studied using bispectral analysis on the data of a fully-kinetic simulation. The nonlinear, in-phase interaction of the oscillations of the azimuthal electric field and the electron density, both tied to the fundamental electron cyclotron drift instability (ECDI) mode, is found to be the main driver of electron transport. Higher-wavenumber ECDI modes do not drive anomalous transport directly; however, they are nonlinearly coupled with each other and with the fundamental ECDI mode. In addition, there is a smaller contribution from a lower-wavenumber mode, not predicted by linear ECDI theory. A reduced model obtained by sparse regression of the data suggests the existence of an inverse energy cascade from the higher ECDI modes to the fundamental one, which would mean that these modes do contribute to transport, albeit indirectly.

## Introduction

Anomalous cross-field electron transport in $\varvec{E} \times \varvec{B}$ plasmas drives the performance losses of many technological devices. In the field of electric propulsion, anomalous transport is known to occur in Hall thrusters [1, 2], but is likely present too in other devices such as electrodeless plasma thrusters [3–5]. In Hall thrusters, anomalous transport significantly increases the electron current to the anode, with respect to the transport caused by electron collisionality alone; in electrodeless plasma thrusters, it likely enhances plasma losses to the walls and plume divergence, resulting in heating and the need to re-ionize the propellant.

The large azimuthal drift velocities in these $\varvec{E} \times \varvec{B}$ plasma discharges are known to give rise to various azimuthal oscillations and instabilities. It is generally agreed that cross-field anomalous transport of electrons stems mainly from correlated oscillations in the electron density $n_e$ and the azimuthal electric field $E_y$, i.e., the time-averaged term $\overline{n_{e} E_{y}}$ in the azimuthal momentum equation [6–8]. The electron cyclotron drift instability (ECDI) has been identified as one of the probable agents giving rise to in-phase oscillations of these quantities, and thus behind the anomalous electron transport [1, 2]. ECDI occurs when the acoustic modes of the ions match the electron cyclotron resonances $m\omega _{ce}$ due to the Doppler shift of the electron $\varvec{E} \times \varvec{B}$ motion. Although the kinetic linear theory of the ECDI is established [9–12] and experimental measurements confirm its existence [13–15], particularly in the setting of Hall thruster plasmas, its nonlinear evolution is still a matter of discussion. In the nonlinear regime, mechanisms such as energy couplings among modes eventually balance the (exponential) instability growth rate, leading to saturation, and an oscillation spectrum quite different from the one predicted from linear theory. Understanding these mechanisms is crucial to unravel the dynamics of anomalous transport during operation, as a first step toward establishing mitigation and control strategies that limit its impact on device performance.

Particle-in-cell (PIC) simulations are a valuable tool to go beyond linear theory and study the saturated nonlinear regime. The seminal PIC simulations of Adam et al. [16] showed the existence of $E\times B$ drift instabilities in a Hall-thruster-like configuration that give rise to in-phase azimuthal oscillations of the electron density and the electric field, which result in axial electron transport. The one-dimensional, azimuthal PIC simulations of Lafleur et al. [8] hinted at an ion-acoustic behavior of the instability, and suggested that saturation occurs due to ion wave trapping. Janhunen et al. [17] found, using a similar approach, that energy in the ECDI excited wave modes is eventually transferred toward long-wavelength, low-frequency modes [17]. These conclusions are in agreement with those of Asadi et al. [18], who also employed a 1D PIC model, showing that the higher wave number modes eventually disappear in steady-state, giving rise to a large wavelength modulation comparable to the entire domain. The results of Tavassoli et al. [19], relying on 1D PIC and a Vlasov codes, also suggest that an inverse energy cascade is present in the nonlinear regime. Although this mechanism has not yet been quantitatively confirmed in more complex simulations, recent experimental work has provided the first direct measurements of both linear growth rates and nonlinear energy transfer associated with the process [15]. Recently, Bello-Benítez et al. [20] used two-dimensional axial-azimuthal PIC simulations to show the development of ion-wave trapping vortexes and that the instability eventually evolves toward a new equilibrium unless ions are renewed from the axial boundaries of the domain, hinting at the importance of 2D aspects for the onset and saturation of the ECDI.

While kinetic simulations offer the raw data to analyze the saturated regime of the ECDI, the characterization of the nonlinear mechanisms that partake in that saturation and the involved energy transfer among wave modes requires specific analysis tools beyond linear Fourier transforms. Bispectral analysis has proven useful to identify nonlinear interactions between plasma oscillation modes in other configurations [5, 21]. The bispectrum (and, its normalized version, the bicoherence) provide a statistical estimate of the phase coupling among three wave modes, e.g. $(\omega _1, \varvec{k}_1)$, $(\omega _2, \varvec{k}_2)$ and the sum $(\omega _1+\omega _2, \varvec{k}_1+\varvec{k}_2)$. A strong nonlinear coupling among a given triad of frequencies and wave numbers yields a large value of the bispectrum.

However, these metrics alone do not permit to determine the direction of the energy flow between the wave modes, which requires establishing differential equations for the power spectral density in each wave mode—including terms for the linear growth/damping rate and three-wave couplings. Nevertheless, the mathematical formulation used to extract the coefficients of such model (see e.g. [15]) relies on solving an ill-posed, overdetermined problem. In this context, alternative, parsimonious approaches such as the Sparse Identification of Non-linear Dynamics (SINDy) technique [22] can offer a more robust framework, as recently demonstrated in a study of Hall thruster breathing mode dynamics [23].

In this paper, we analyze the nonlinear dynamics present in the 2D PIC simulation data of an $\varvec{E} \times \varvec{B}$ discharge. The chosen geometry is analogous to that of a Hall thruster discharge channel, and retains the axial and azimuthal directions, which allows capturing the key features of the ECDI in these devices. The simulation is carried out using the model of Bello-Benítez et al. [20]. Our analysis focuses on the part of the macroscopic axial electron current, $j_{ze}$, driven by in-phase azimuthal oscillations of the electron density $n_e$ and the electric field $\varvec{E}$. Firstly, bispectral analysis directly allows us to link the observed wave spectrum with anomalous transport, quantifying the direct contribution of each mode to $j_{ze}$. This shows that the dominant mechanism for cross-field electron transport are the in-phase density-field fluctuations associated with the first mode of the ECDI, rather than other higher-order modes. Secondly, we employ bispectral analysis to demonstrate that the multiple, unstable ECDI modes are interconnected through an inverse energy cascade. Since this approach alone does not fully determine the energy source in the fluctuations or the direction of energy transfer between modes, we apply sparse-regression data-driven modeling with SINDy to a plasma slab to track energy evolution at dominant frequencies. Our findings reveal that quadratic three-wave coupling can explain at least part of the energy flow among wave modes via an inverse energy cascade, from higher-wavenumber modes to spectral bands responsible for cross-field transport.

The rest of the paper is structured as follows: Simulation overview provides a brief overview of the simulation and the data used to carry out this work; the Methods section introduces the techniques used to analyze and model the data; Results and discussion outlines the results obtained, divided into Spectral analysis of the discharge for the identification of the different modes present in the discharge, Anomalous axial transport for the quantification of dominant contributions to the anomalous electron current, and Three-wave power coupling for the reduced models of power transfer based on nonlinear three-wave coupling. Finally, Summary gathers the conclusions of the study. The reader is advised to check the previous work [20], since the theory, simulations, and fundamental structure of the observed fluctuations, are described there. A preliminary version of this work was presented in the 38th International Electric Propulsion Conference [24].

## Simulation overview

This work analyzes a canonical Hall thruster-like $\varvec{E} \times \varvec{B}$ plasma discharge by means of simulation data, obtained using the in-house, 2D, electrostatic PIC code named PICASO [20, 25]. The simulations describe the collisionless ion and electron dynamics in an axial-azimuthal (*z*, *y*) domain, with a radial applied magnetic field and axially streaming ions. The simulation conditions are representative of a section of a Hall thruster discharge channel away from anode, cathode and walls. The details of the code are given in those references, and in particular, we use the simulation setup of [20]. Nevertheless, the essential details are described next for self-completeness. In essence, the code implements the standard PIC scheme, where the equations of motion of the ion and electron macroparticles are solved with an explicit, momentum-conserving Boris algorithm and the interpolation and weighting schemes implement first-order bi-linear shape functions. The Poisson solver used here employs second-order finite-differences to discretize the Laplace operator and PARDISO Intel MKL direct solver to invert the resulting linear system. The code is implemented in Fortran90 and the operations on macroparticles are parallelized following a particle-decomposition strategy using shared-memory OpenMP. PICASO has been thoroughly verified internally using simple test cases, and cross-verified against other PIC codes as part of the 2D PIC Penning discharge international benchmark [26].

**Fig. 1:**
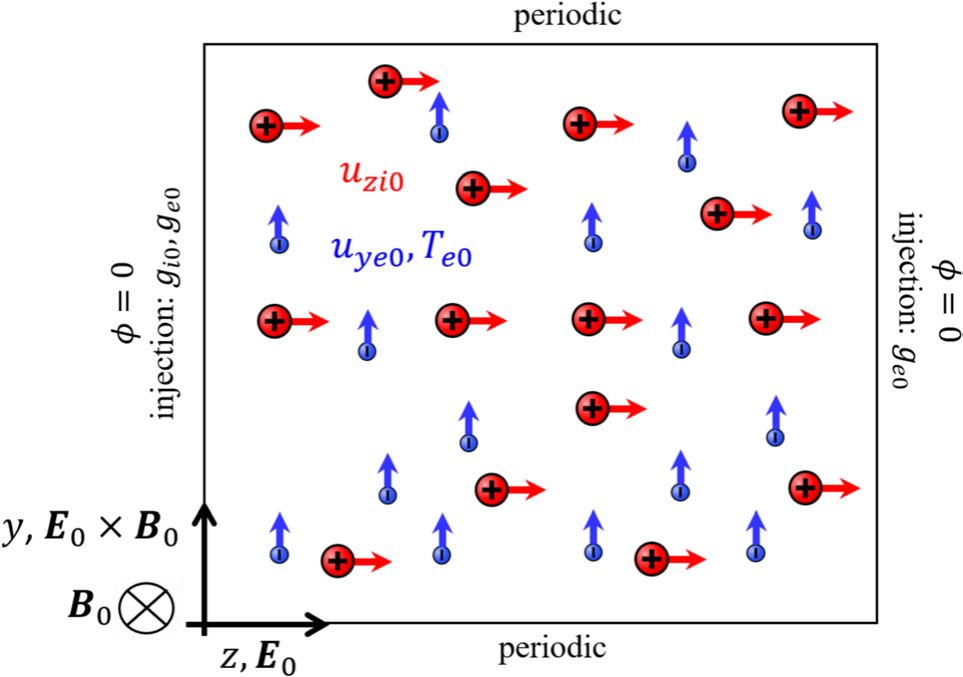
Diagram sketching the full-PIC simulation domain, boundary conditions and initial equilibrium state

Figure 1 sketches the simulation setup. The simulation solves the plasma oscillations in a rectangular domain in the axial ($z=[0,L_z]$) and azimuthal ($y=[0,L_y]$) directions, with periodic conditions along the *y* boundaries. A uniform, out-of-plane magnetic field $\varvec{B} = B_0\varvec{1}_x$ is imposed, as well as an axial electric field $\varvec{E}_0=E_0\varvec{1}_z$.

The simulation starts from the initial equilibrium configuration used in linear ECDI studies, namely, a uniform quasineutral plasma with electrons drifting azimuthally with a velocity $u_{ye0} \varvec{1}_y$ corresponding to the $E\times B$ drift induced by the applied fields, $u_{ye0} = E_0/B_0$, while ions drift axially with $u_{zi0} \varvec{1}_z$, for a chosen value of $u_{zi0}$. The values of these properties and the rest of physical and numerical parameters are gathered in Table 1; this simulation case represents a typical operating condition for a Hall thruster and was chosen from a set of cases because it prominently exhibits the instability, as highlighted in a previous parametric study [20]. The spontaneous development of instabilities sets up a perturbing electrostatic field, $\varvec{E}_1=-\nabla \phi$, which affects the dynamics of electrons and ions. The electrostatic potential $\phi$ is computed by solving the Poisson equation with periodic boundary conditions along the *y* borders of the domain and Dirichlet conditions along the *z* boundaries.

**Table:** Physical and numerical parameters of the reference simulation case

Description and symbol	Value and units
Ion mass, $m_i$	1 amu
Applied electric field, $E_0$	$10^4$ V/m
Applied magnetic field, $B_0$	200 G
Plasma density, $n_0$	$10^{17}$ m$^{-3}$
Ion axial velocity, $u_{zi0}$	10 km/s
Initial electron temperature, $T_{e0}$	6 eV
Azimuthal domain length, $L_y$	5.359 mm
Axial domain length, $L_z$	2.679 mm
$E_0\times B_0$ drift, $u_{ye0}$	500 km/s
Electron thermal speed, $c_{e0}$	1027 km/s
Ion sound speed, $c_{s0}$	23.97 km/s
Debye length, $\lambda _{D0}$	57.58 $\mu$m
Electron Larmor radius, $\rho _{e0}$	292.0 $\mu$m
Electron plasma frequency, $\omega _{pe0}$	2.839 GHz
Electron gyrofrequency, $\omega _{ce}$	0.5600 GHz
Ion plasma frequency, $\omega _{pi0}$	66.26 MHz
Lower-hybrid frequency, $\omega _{lh}$	13.07 MHz
Number of cells in *y* direction, $N_y$	100
Number of cells in *z* direction, $N_z$	50
Number of particles per cell, $N_{ppc }$	$\sim 200$
Time step, $\Delta t$	$5\cdot 10^{-12}$ s
Print-out time step, $\Delta t_{print}$	$10^{-9}$ s
Total simulation time, $t_{final}$	$50\cdot 10^{-6}$ s
Cell size, $\Delta y$, $\Delta z$	53.59 $\mu$m

The subscript ‘0’ stands for initial equilibrium conditions. Derived parameter values are included for completeness

Following the same approach of other PIC studies on the ECDI [17, 19, 20] and of the ideal linear theory [9, 10], we ignore the effects of the background magnetic and electric fields on the ion dynamics. This approximation of unmagnetized and unaccelerated ions, which is acceptable in the limit of small $eB_0/m_i$ and $E_0L_z/(m_iu_{zi0}^2)$, is necessary to guarantee that the initial conditions prescribed above be an equilibrium configuration. Crucially, this simplification does not affect the development of the ECDI, which depends mainly on the electron drift and the $\varvec{E}_1$ fluctuation field. Previous work [20] discusses in detail the finer implications of this approach.

Initially, electrons are randomly sampled from a drifting Maxwellian velocity distribution function with density $n_0$, temperature $T_{e0}$, and velocity $u_{ye0} \varvec{1}_y$. Ions are sampled from a cold, drifting distribution with $n_0$ and $u_{zi0} \varvec{1}_z$. Particles can cross freely along the periodic *y* boundaries of the domain. Any particles reaching the *z* boundaries of the domain are removed from the simulation. To replenish the domain, the same distributions as above are used to inject new particles from the *z* boundaries: ions are injected through the $z=0$ boundary with flux density $n_0 u_{zi0}$, and electrons are injected through the $z=0,L_{z}$ boundaries with thermal fluxes $\pm n_0 c_{e0}/\sqrt{2 \pi }$, where $c_{e0}=\sqrt{T_{e0}/m_e}$. This replenishment is crucial to maintain the discharge over time, and to mimic as close as possible the conditions of a section of the plasma discharge away from the upstream anode and the downstream cathode. Indeed, switching off the replenishment at the boundaries (which would correspond to the conditions in the presence of a wall) would result in the formation of strong sheaths at the edges of the domain, caused by the removal of escaping electrons, and the eventual extinction of the discharge.

At the initial equilibrium state, the axial current of electrons, $j_{ze0}$, is zero. Since collisions between particles are disregarded, any subsequent electron axial current is due to the cross-field transport generated by plasma oscillations. As the simulation proceeds and the instability develops and eventually saturates, in-phase azimuthal oscillations of density and potential cause the generation of a nonzero $j_{ze0}$. The electron distribution function also evolves with evident electron heating, reaching a final temperature of about 8–10 eV. Because of the discrete nature of the simulation domain in the *y* direction, the possible wavenumbers are limited to integer multiples of $L_y$. By choosing $L_y=6\cdot 2\pi {u_{ey0}}/{\omega _{ce}}$, the allowed wavenumbers become $k_{}={n\omega _{ce}}/{6u_{ey0}}$. The chosen discretization ensures that the modes $k_{}L_y/2\pi =n=1+6m$ $(m=1,2,3...)$ align closely with the peaks in linear growth rate predicted by ECDI theory, corresponding to successive cyclotron harmonic resonances [20].

Compared to the original study in [20], the present simulation shares the exact same setting but was run for 50 $\mu s$ to achieve better statistics in our analysis and validate the original results. Regarding the kinetic and macroscopic variables to be analyzed, they are sampled from the output of the simulation at multiples of $\Delta t_{print} = N_{print}\Delta t$. The initial transient stage of the simulation is disregarded, and we keep times from 1$\mu s$ to 50$\mu s$ in which the instability has already reached nonlinear saturation and the spectrum is statistically weakly-stationary.

## Methods

In the following, we first introduce the spectral quantities, and then the formalism behind the sparse identification of nonlinear dynamics (SINDy) technique, employed in the reminder of the paper.

### Spectral and bispectral analysis

A signal $x_a$ sampled over a spatiotemporal domain may be described in terms of a collection of discrete Fourier modes:

1 $$\begin{aligned} x_a(t_i, y_j) = \sum \limits _m \hat{x}_a(\omega _m, y_j)e^{-\text {i}\omega _mt_i} = \sum \limits _n \tilde{x}_a(t_i,k_n)e^{\text {i} k_ny_j} = \sum \limits _n\sum \limits _m \hat{\tilde{x}}_a(\omega _m, k_n)e^{-\text {i}\omega _mt_i+\text {i} k_n y_j} \end{aligned}$$

where $\text {i}$ is the imaginary unit and $\omega _m$ and $k_n$ cover both positive and negative frequencies with the complex conjugate property that $\hat{x}_a^{*}(-\omega _m, y_j)=\hat{x}_a(\omega _m, y_j)$, $\tilde{x}_a^{*}(t_i, -k_n)=\tilde{x}_a(t_i, k_n)$, and $\hat{\tilde{x}}_a^{*}(-\omega _m, -k_n)=\hat{\tilde{x}}_a(\omega _m, k_n)$.

Given two signals $x_a(t_i, y_j)$ and $x_b(t_i, y_j)$, the second-order cumulant spectrum, also known as the (cross) power spectral density (CPSD), is defined as:

2 $$\begin{aligned} P_{ab}(\omega _m,k_n) = \left\langle \hat{\tilde{x}}_a(\omega _m, k_n)\hat{\tilde{x}}^{*}_b(\omega _m, k_n)\right\rangle . \end{aligned}$$

When signals $x_a$ and $x_b$ are the same signal, $P_{aa} \equiv P_a$, and we simply refer to this as the power spectral density (PSD). Here, $\left\langle \cdot \right\rangle$ denotes averaging over multiple independent instances (realizations). In our case, these realizations correspond to separate time intervals (windows) from the same simulation, assuming a statistically weakly-stationary process. All averaged quantities in this work have been computed taking windows of 0.5 $\mu$s with $50\%$ overlap, unless otherwise indicated. After removing the initial 1 $\mu$s transient in our 50 $\mu$s simulation, this results in a total of 195 temporal windows. A sensitivity analysis confirmed that our conclusions are unaffected by this choice of parameters.

Summing over all $k_n$ modes we can also define

3 $$\begin{aligned} P_{ab}(\omega _m) = \sum \limits _n P_{ab}(\omega _m,k_n) = \frac{1}{N_j}\sum \limits _j\left\langle \hat{{x}}_a(\omega _m, y_j)\hat{{x}}^{*}_b(\omega _m, y_j)\right\rangle , \end{aligned}$$

where $N_j$ denotes the number of points in the *y* direction. Here, we have used $\hat{x}(\omega _m, y_j) = \sum \limits _n \hat{\tilde{x}}(\omega _m, k_n) e^{\text {i} k_n y_j}$ and the orthogonality properties of the discrete Fourier transform. Similarly, we can define $P_{ab}(k_n)$ summing over all frequencies $\omega$.

Linear spectral analysis, such as the use of Eq. 2, is limited when spectral components interact nonlinearly, resulting in energy transfer between modes [27]. These nonlinear interactions are not captured by the PSD alone, making it insufficient for fully characterizing the dynamics. Higher-order spectral techniques can reveal the existence of stable phase-locking relations among the Fourier modes of a signal, helping identify these nonlinear couplings. For the lowest order (i.e., quadratic) nonlinearities, three-wave coupling can occur if the resonance conditions,

4 $$\begin{aligned} \omega _1+\omega _2 =\omega _3,\qquad \qquad \quad k_1 + k_2 =k_3, \end{aligned}$$

are satisfied among three propagating modes ($\omega _1$, $k_1$), ($\omega _2$, $k_2$) and ($\omega _3$, $k_3$). Given three signals $x_a(t_i, y_j)$, $x_b(t_i, y_j)$, $x_c(t_i, y_j)$, we define their (cross) bispectrum as the third-order cumulant spectrum [28],

5 $$\begin{aligned} \mathfrak {B}_{abc}(\omega _m, \omega _l, k_n, k_p) = \left\langle \hat{\tilde{x}}_a(\omega _m, k_n)\hat{\tilde{x}}_b(\omega _l, k_p)\hat{\tilde{x}}^*_c(\omega _m+\omega _l, k_n+k_p) \right\rangle . \end{aligned}$$

Reduced versions of the bispectrum can be defined in terms of only the frequencies or the wavenumbers, analogous to the reduced versions of the PSD. For example, in terms of frequencies only we define, by summing over all $k_n$, $k_p$ modes,

6 $$\begin{aligned} \mathfrak {B}_{abc}(\omega _m, \omega _l) =\sum \limits _n\sum \limits _p\mathfrak {B}_{abc}(\omega _m, \omega _l, k_n, k_p) = \frac{1}{N_j}\sum \limits _j \left\langle \hat{x}_a(\omega _m, y_j) \hat{x}_b(\omega _l, y_j)\hat{x}^*_c(\omega _m+\omega _l,y_j) \right\rangle . \end{aligned}$$

The latter equality follows, again, from the orthogonality properties of the discrete Fourier transform. The reduced $\mathfrak {B}_{abc}(k_n, k_p)$ can be similarly defined.

From the bispectrum, the normalized bicoherence is defined as

7 $$\begin{aligned} b_{abc}(\omega _m, \omega _l, k_n, k_p) = \frac{\left| \mathfrak {B}_{abc}(\omega _m, \omega _l, k_n, k_p) \right| }{\sqrt{\left\langle \left| \hat{\tilde{x}}_a(\omega _m, k_n) \hat{\tilde{x}}_b(\omega _l, k_p) \right| ^2 \right\rangle \left\langle \left| \hat{\tilde{x}}^*_c(\omega _m+\omega _l, k_n+k_p) \right| ^2 \right\rangle }}. \end{aligned}$$

Similarly to what has been shown with the bispectrum, $b_{abc}(\omega _m, \omega _l)$ and $b_{abc}(k_n, k_p)$ can be defined. When $x_a$, $x_b$, and $x_c$ are the same signal, one speaks simply of $b_{aaa}\equiv b_{a}$ as the (self-)bicoherence of that signal.

Phase-locked modes, and hence quadratic couplings, result in a value of $b_a$ close to 1, whereas bicoherence values close to 0 are indicative of random phases or noise. Specifically, the 95% significance level for null bicoherence computed over *N* realizations is [29] $\sqrt{3/N}$. For the reduced $b_{abc}(\omega _m, \omega _l)$, taking into account the temporal windows (195) and the number of azimuthal points (100), the actual number of realizations is $N=19500$, corresponding to a significant level above $b_a=0.012$. Discrete interactions between two modes show up as “islands” of high bicoherence, whose width corresponds to the spectral broadening of interacting peaks. Continuous interactions of a single frequency with a broader band are shown as lines or segments, either verticals ($\omega _1=\text {const}$), horizontals ($\omega _2=\text {const}$) or diagonals ($\omega _3=\text {const}$). Note that a high bicoherence correlates statistically with, but does not causally ensure, a strong quadratic phase coupling among modes. Moreover, bicoherence itself does not discriminate the direction of power flow among modes, and this directionality needs to be studied by other methods, such as by fitting the underlying three-wave coupling equations.

The self-bicoherence of a real signal features a number of symmetries. Focusing on the reduced $b_a(\omega _m,\omega _l)$, i.e. the one depending only on the two frequencies, we have e.g.

8 $$\begin{aligned} b_{a}(\omega _m, \omega _l) = b_{a}(\omega _l, \omega _m) = b_{a}(\omega _m, -\omega _m-\omega _l) = b_{a}(-\omega _m, -\omega _l), \end{aligned}$$

which mean that all information of $b_a$ is contained in a triangular sector in the first quadrant of the $(\omega _m,\omega _l)$ plane [28]. In practice, the region in which $b_a$ is computed is further reduced to a finite-size triangle once the Nyquist limit $\omega _m, \omega _l, \omega _m+\omega _l < \omega _s/2$ for a sampling frequency of $\omega _s$ is taken into account.

### Sparse Identification of Nonlinear Dynamics (SINDy)

The basic form the SINDy framework [22] operates as follows. We consider a dynamical system given by a state vector $\varvec{x}(t) = \left[ x_1(t),x_2(t),\ldots ,x_I(t)\right] ^T$ in a state space $\mathbb X$, governed by a set of ordinary differential equations of the form

9 $$\begin{aligned} \dot{x}_i(t)=f_i(\varvec{x},t), \end{aligned}$$

where $f_i$ ($i=1,\ldots ,I$) are unknown functions of the state, and possibly, time. We aim to write each $f_i$ in (9) as

10 $$\begin{aligned} f_i(\varvec{x},t) = \beta _{ij} \Theta _j (\varvec{x},t), \end{aligned}$$

where $\Theta _j$ ($j=1,\ldots , J$) is a chosen collection of functions (termed “features”) and $\beta _{ij}$ a (sparse) array of coefficients to be determined.

If a realization of the dynamical system has data $x_i(t_k) \equiv x_{ik}$ at discrete time instants $t_k$ ($k=0,\ldots , K$), potentially subject to noise, it is possible to estimate the coefficients $\beta _{ij}$ from the following linear system of equations:

11 $$\begin{aligned} \dot{{x}}_{ik} = \beta _{ij}\Theta _{jk} \end{aligned}$$

where $\dot{{x}}_{ik}$ is a numerical estimate of the time derivatives of the state from the data, using e.g. finite differences, and $\Theta _{jk}\equiv \Theta _j({\varvec{x}}(t_k), t_k)$.

This set of equations is typically strongly overdetermined, as we have many more equations than unknown coefficients, $K \gg IJ$. A first, naive approach is to solve for $\beta _{ij}$ by minimization of the least-square errors

12 $$\begin{aligned} \varepsilon ^{S}_i= \frac{1}{N}\frac{1}{{\sigma }^2_{i}}\sum \limits _{k} \left( \dot{{x}}_{ik} - \beta _{ij}{\Theta }_{jk} \right) ^2, \quad i=1,\ldots ,I, \end{aligned}$$

where *N* stands for the sample size and ${\sigma }^2_{i}$ for the variance of the numerical derivatives of the *i*-th variable, ${x}_{ik}$. An error $\varepsilon ^S_i$ close to zero indicates that the model closely reproduces the numerical derivatives of the data. However, minimizing $\varepsilon ^{S}_i$, $i=1,\ldots ,I$ directly typically yields a full $\beta _{ij}$ matrix, with most coefficients different from zero. This is usually undesired, as the resulting models are highly sensitive to noise, exhibit an unaffordable complexity and lack simple physical interpretation. What SINDy proposes instead is finding $\beta _{ij}$ by minimizing the sum of the least squares errors $\varepsilon ^{S}_i$, plus a sparsity-promoting regularization term (or penalty) $\varepsilon _i^\lambda$,

13 $$\begin{aligned} \beta _{ij} = \underset{\beta _{ij}}{\text {argmin}}\ {\left( \varepsilon ^{S}_i+\varepsilon ^\lambda _i\right) }. \end{aligned}$$

By regularizing to promote sparsity in the solution $\beta _{ij}$, the algorithm is shown to regress on the features most relevant to the dynamics and discard the rest [22]. In the present work, we use the ALASSO penalty [30, 31]

14 $$\begin{aligned} \varepsilon ^{\lambda }_i=\left| \sum \limits _j a_{ij}\beta _{ij} \right| \quad \text {with }a_{ij}=\frac{\lambda _i}{\beta _{ij}^*} \quad (\text {no sum in}\ i), \end{aligned}$$

where the $\lambda _i$ are hyperparameters which set the relative weight of the regularization term over the error term for each state variable, and the term-specific weights $\beta _{ij}^*$ are the coefficient estimates coming from optimizing $\varepsilon ^S_i$ alone,

15 $$\begin{aligned} \beta _{ij}^* = \underset{\beta _{ij}}{\text {argmin}}\ {\left( \varepsilon ^{S}_i\right) }. \end{aligned}$$

This form of $\varepsilon ^\lambda _i$ puts a large penalty on small coefficients while reducing biases on the larger coefficients [31]. The ALASSO penalty also has the benefit of leading to a computationally-efficient convex minimization problem, and offers consistent variable selection and correct coefficient estimation as the number of samples *K* tends to infinity (given that all relevant features are included in the chosen function library, and available data spans the whole state space sufficiently).

For each state variable $x_i$, sweeping the regularization parameter $\lambda _i$ from 0 to infinity will yield different models, or coefficients $\beta _{ij}$. Plotting the error of each model against the number of non-zero terms in $\beta _{ij}$ results in an L-shaped *Pareto front*, from which the optimal model can be selected based on the knee inflection point [23, 32] or previous knowledge of the system. While the model at the Pareto front knee can be deemed the best-performing with the fewest terms, previous studies [33] have shown that when the library of candidate terms is guided by fundamental physics and domain expertise, the entire Pareto front represents a hierarchy of progressively simplified models. These models successively approximate the full physical equations, with each step adding the most dynamically significant terms, which is valuable information as to their relative importance that we leverage in this work.

In the search for reduced models that explain the quadratic couplings in the spectra of a set of signals $x_i$ (for $i=a,b,c,\ldots$), we compute their PSD $P_i(\omega _m,k_n;t_k)$ on a sliding time window centered on $t_k$ (i.e., the spectrogram), and apply the SINDy algorithm to retrieve the approximate evolution equation for the $P_i$. Observe that, for our purpose, the choice of the spectral Fourier data decomposition in $(\omega _m, k_n)$ is a natural one, as it yields PSDs per frequency and per wavelength, which is exactly what is needed for our analysis. We note in passing that other decomposition methods, such as Proper Orthogonal Decomposition (POD) or Dynamic Mode Decomposition (DMD), have also been successfully applied to analyze oscillations in $\varvec{E} \times \varvec{B}$ plasma simulations [34–36] in other works.

## Results and discussion

### Spectral analysis of the discharge

**Fig. 2:**
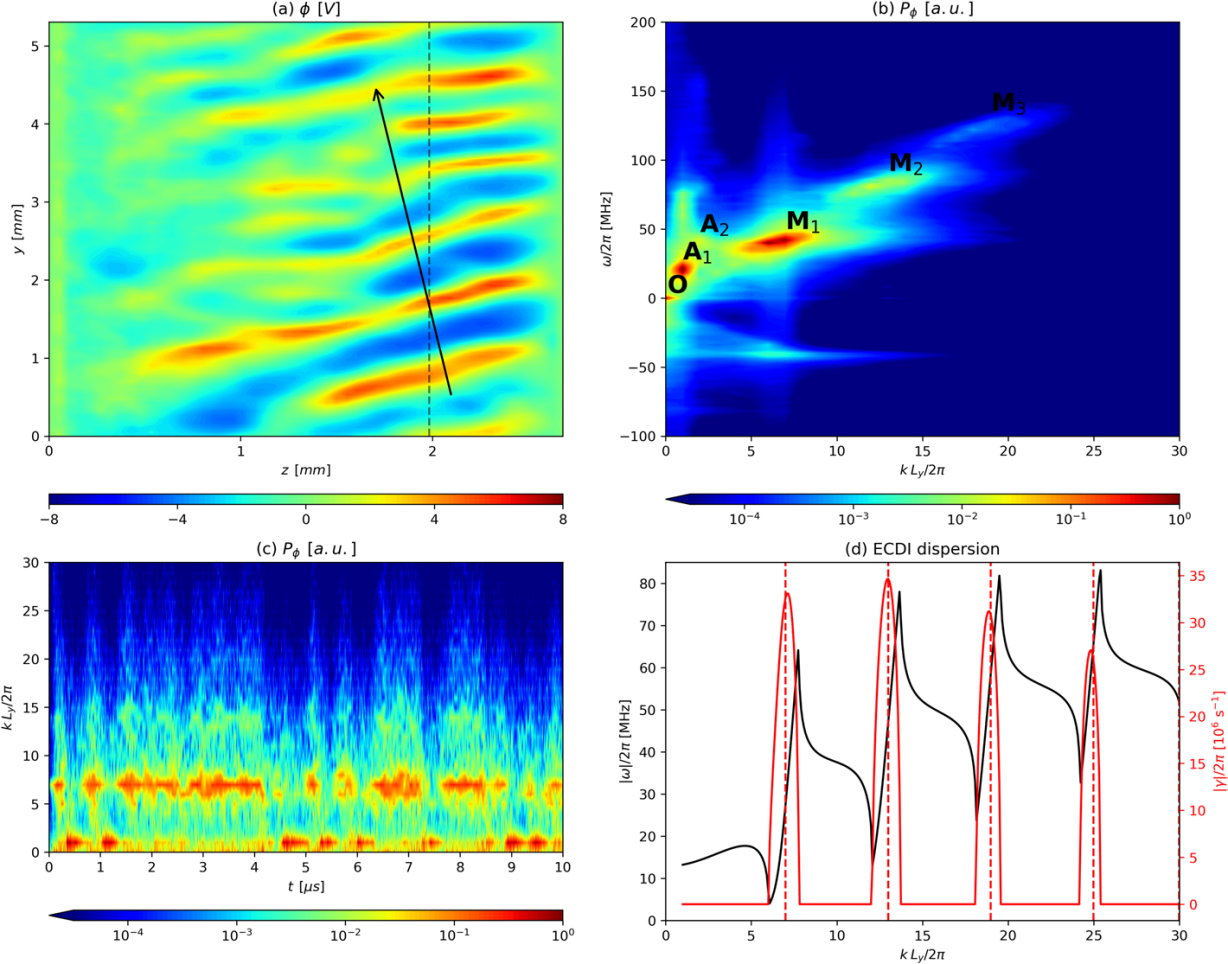
Oscillations of the electrostatic potential $\phi$ present in the full-PIC simulation. **a** Snapshot of $\phi$ at $t=2.45$ $\mu$s; **b** PSD dispersion diagram in the $\omega$–*k* plane at $z=1.98$ mm; **c** PSD *k*–*t* spectrogram at $z=1.98$ mm; **d** Real frequency and growth rate, respectively in black and red, as computed from linear theory [20]. The vertical dashed red lines mark $kL_y/(2\pi )=7,13,19$, the centroids of the *M* modes observed in (**b**). The dotted black line in (**a**) denotes the axial slice ($z=1.98$ mm) where both the dispersion diagram and spectrogram are computed, while the black arrow displays the propagation direction of the oscillations. Plots (**b**), and (**c**) have been normalized by dividing each by its respective maximum value

In previous work [20], short wavelength unstable modes were reported to grow from the initially homogeneous plasma, quickly evolving into a nonlinear stage and then saturating, reaching a new state that also featured long wavelength modes. Taking the perturbation electrostatic potential $\phi$ as a representative variable of the plasma behavior, a snapshot of it is shown on Fig. 2a.

The oscillations are stronger in the downstream part of the domain and propagate in the direction of the arrow, i.e. primarily in the $+y$ direction, with a minor component of the wave vector along the $-z$ direction. The analysis in this work focuses mainly on data in an differential axial slab in this region, at $z =1.98$ mm, shown in Fig. 2a, where the electrostatic oscillations are large.

The discrete Fourier transform on *t* (for the full span of the simulation minus the initial transient) and on *y* (on this axial slice) are shown on Fig. 2b. Please note that we use the symbol *k* to denote the wavenumber along the *y* direction. This likely means that most of the spectral power is indeed in modes traveling in the $+y$ direction, although there is some power in modes traveling in the $-y$ direction as well. As most fluctuation power is located in the first quadrant (Fig. 2b), our subsequent analysis focuses on the $\omega k>0$ part of the spectrum.

Six prominent peaks can be identified in $(\omega /(2\pi ),k/(2\pi ))$ space, given in MHz and multiples of $1/L_y$, respectively, which we shall label as modes $M_1=(42,7)$, $M_2=(84,13)$, and $M_3=(126,19)$; modes $A_1=(20,1)$, $A_2=(40,2)$, and mode $O=(1.6,0)$. Note that the high value of the frequencies (compared to those typical of Hall thrusters) results from using 1 amu ions in the simulation instead of the usual, heavier propellants; a discussion of the scaling of the mode frequencies with the mass can be found in [20].

The *M* modes constitute a branch of comparatively broadband harmonics with an associated phase velocity of about 32 km/s, while the *A* modes have roughly 107 km/s. This is, respectively, 1.3 and 4.5 times the ion sound speed computed in Table 1. The lone mode *O* is a near-axial mode that appears to modulate the other modes, in particular those of the *M* branch. The axial character of mode *O* and the proximity of its frequency to the ion transit frequency in the domain, $u_{zi}/L_{z}=0.9$ MHz from Table 1, hint at the possible relation of this mode with the (slow) axial ion dynamics.

The modes observed in the spectrum of $\phi$ also appear in the spectra of other plasma variables, though with varying relative amplitudes. In particular, we verify that the spectrum of $E_y=-\partial \phi /\partial y$ of interest in the subsequent sections mirrors that of $\textrm{i} k\phi$. The *k* factor in this expression enhances the importance of mode $M_3$ in the spectrum of $E_y$, which otherwise seems weak in the spectrum of $\phi$.

Figure 2c showcases the spectrogram of $\phi$, i.e., the time evolution of the azimuthal modes in *k* space. The periods of high amplitude in the modes of the *M*-branch alternate in time with those of the *A*-branch, modulated by a low frequency compatible with mode *O*. Like $\phi$, other plasma properties and the anomalous current oscillate in time, alternating between periods of growth and quenching.

Figure 2d displays linear frequency $\omega$ and growth rates $\gamma$ of the first ECDI modes versus the azimuthal *k* number as predicted by linear theory [9, 20]. The wavenumber of the peaks of modes $M_1$ through $M_3$ in Fig. 2b show a good agreement with the linear theory *k*, indicating that the modes of the *M* branch correspond with the first ECDI modes. Nevertheless, it is evident that there is significant spectral broadening of the modes at saturation.

In contrast, the frequencies of the ECDI modes in Fig. 2b deviate substantially from linear theory predictions of Fig. 2d. Deviation from the linear frequencies is expected once nonlinear saturation disrupts the assumed coherent modes and initially-Maxwellian distributions [17]. Similarly, a naive interpretation of Fig. 2d would lead us to assume that mode $M_2$ should exhibit the largest amplitude, as it features a greater linear growth rate $\gamma$ than the other modes. Notwithstanding this, the PSD in Fig. 2b reveals that actually mode $M_1$ dominates in terms of amplitude/energy with respect to the other *M* modes. This is a first hint that nonlinear interactions cause the transfer of energy toward the lowest ECDI mode, $M_1$. Incidentally, despite the evident peak broadening in Fig. 2b, discreteness of the modes persists, unlike the continuous dispersion reported in other studies of the ECDI’s later evolution [8, 12, 14].

Remarkably, the *A* modes and the *O* mode are not predicted by the linear ECDI theory, which indicates that they are due to either other linear instability mechanisms or result from nonlinear excitation by the other modes. The analysis in Three-wave power coupling section further explores this possibility.

### Anomalous axial transport

In the previous section, we identified several oscillation modes that are present in the electrostatic potential $\phi$ (and which appear similarly in other plasma variables). We now aim to determine how each mode affects cross-field transport, giving rise to an axial electron current, $j_{ze}=-en_e\varvec{u}_e$.

A single electron (macroparticle) with position and velocity $\varvec{r}_e$, $\varvec{v}_e$ and subject to the fields $\varvec{E} = E_0\varvec{1}_z+\varvec{E}_1$, $\varvec{B} = B_0\varvec{1}_x$ has an instantaneous gyrocenter position defined as

16 $$\begin{aligned} \varvec{r}_g = \left( z_e + \frac{m_ev_{ye}}{e B_0}\right) \varvec{1}_z + \left( y_e - \frac{m_ev_{ze}}{e B_0}\right) \varvec{1}_y, \end{aligned}$$

whose rate of change is given by the zeroth-order drift $u_{ye0}=E_0/B_0$ and the time-varying drift induced by $\varvec{E}_1\times \varvec{B}$,

17 $$\begin{aligned} \frac{\textrm{d} \varvec{r}_g}{\textrm{d} t} \equiv \varvec{v}_g = u_{ye0} \varvec{1}_y -\frac{E_{y1}}{B_0}\varvec{1}_z + \frac{E_{z1}}{B_0}\varvec{1}_y. \end{aligned}$$

In the limit of negligible electron Larmor radius, $\ell _e\rightarrow 0$, the electron current $\varvec{j}_{e}$ equals the gyrocenter current, equal to $-en_e\varvec{v}_g$. Hence, the axial electron current is due solely to the gyrocenter drift induced by the $E_{y1}$ field. We shall denote this current as

18 $$\begin{aligned} j_{ze}^{E}(t, y)\equiv en_e\frac{E_{y1}}{B_0} \end{aligned}$$

As the azimuthal average of $E_{y1}$ is zero, there can only be a net azimuthally-averaged axial flux of electrons if the product $n_e E_{y1}$ does *not* average to zero. This requires in-phase fluctuations of the electron density $n_e$ and the field $E_{y1}$.

**Fig. 3:**
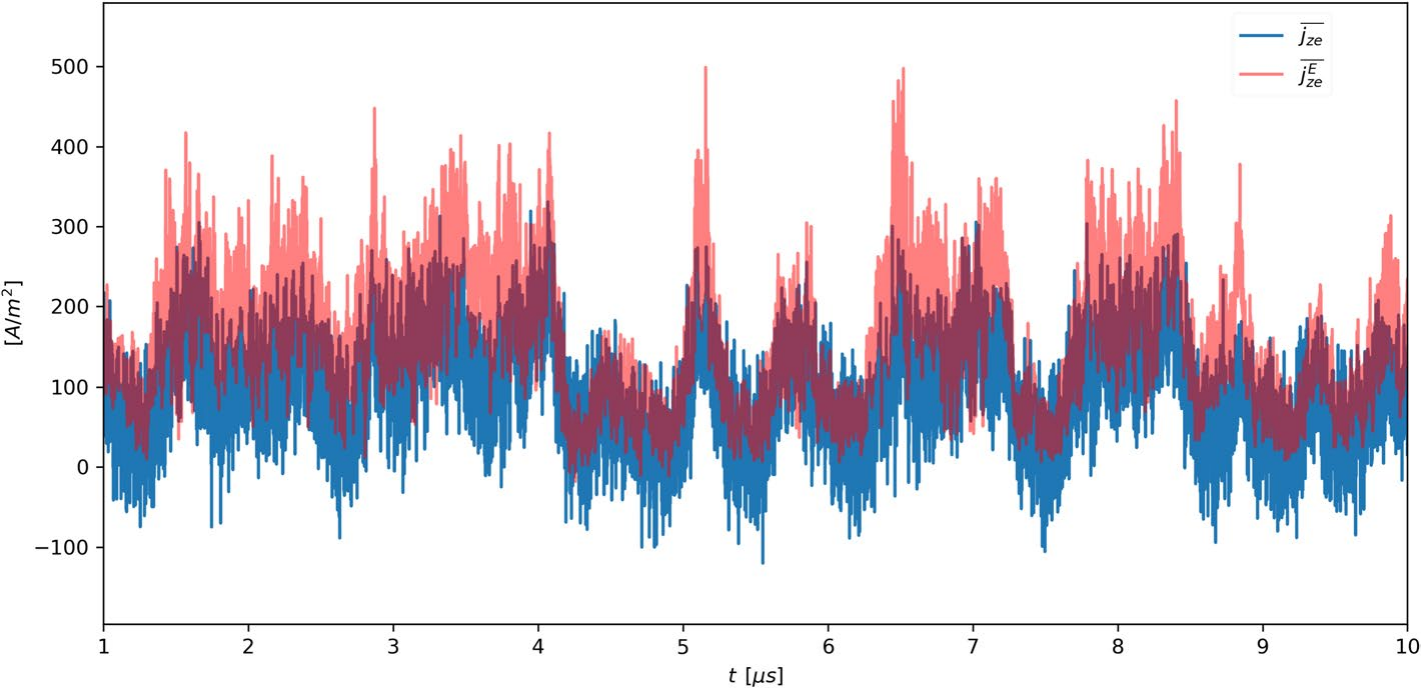
Time evolution of the *y*-averaged electron axial current $j_{ze}$ (i.e., its $k=0$ component) and the $j_{ze}^{E}$ current caused by the correlated $n_e$, $E_y$ fluctuations, at $z=1.98$ mm

For an electron population with a finite characteristic electron Larmor radius $\ell _{e0} = \sqrt{m_eT_{e0}}/(eB_0)$, the macroscopic electron current $\varvec{j}_e$ can have additional contributions. In particular, the axial component of this current, given by the azimuthal component of the collisionless electron momentum equation, is

19 $$\begin{aligned} j_{ze}(t, y)= en_e \frac{E_{y1}}{B_0} -\frac{m_e}{B_0} \left( \frac{\partial }{\partial z}M_{zy e}+\frac{\partial }{\partial y}M_{yy e}+\frac{\partial }{\partial t} n_eu_{ye}\right) = j_{ze}^{E}(t,y) + O\left( en_{e0}c_{e0}\frac{\ell _{e0}}{L_z}\right) , \end{aligned}$$

where $L_z$ is taken as the relevant gradient length, $M_{zy e}$ and $M_{yy e}$ are the axial-azimuthal and azimuthal components of the electron momentum tensor, $M_e=\int \int \varvec{v}_e \varvec{v}_e f_ed^3\varvec{v}_e$, and radial dynamics have been disregarded. The last term on the right hand side is a gyroviscous/inertial finite-Larmor-radius effect, and is small as long as $\ell _{e0}/L_z$ is small. Indeed, in our simulation this term only drives the axial current dynamics near the axial edges of the domain, in a layer of characteristic thickness comparable to $\ell _{e0}$, where electron gyro-orbits are interrupted by the domain edge. This is illustrated in Fig. 3 at our chosen analysis location, $z=1.98$ mm, where the azimuthally-averaged macroscopic electron current $\overline{\jmath _{ze}}$ is seen to follow the gyrocenter flux $\overline{\jmath ^E_{ze}}$ and displays similar fluctuations. At this location, the gyroviscous/inertial term has a generally negative contribution to macroscopic current.

The primary interest of anomalous transport studies is determining the azimuthally-averaged gyrocenter flux $\overline{\jmath _{ze}^E}$, i.e., the one corresponding to azimuthal mode $\omega =0$ and $k=0$, as this is the one associated with a net transport of electrons in the axial direction. Notwithstanding this, it is also relevant to characterize $\omega \ne 0$ and $k\ne 0$ components of $j_{ze}^E$, as this can yield additional insight on the dynamics of the problem.

**Fig. 4:**
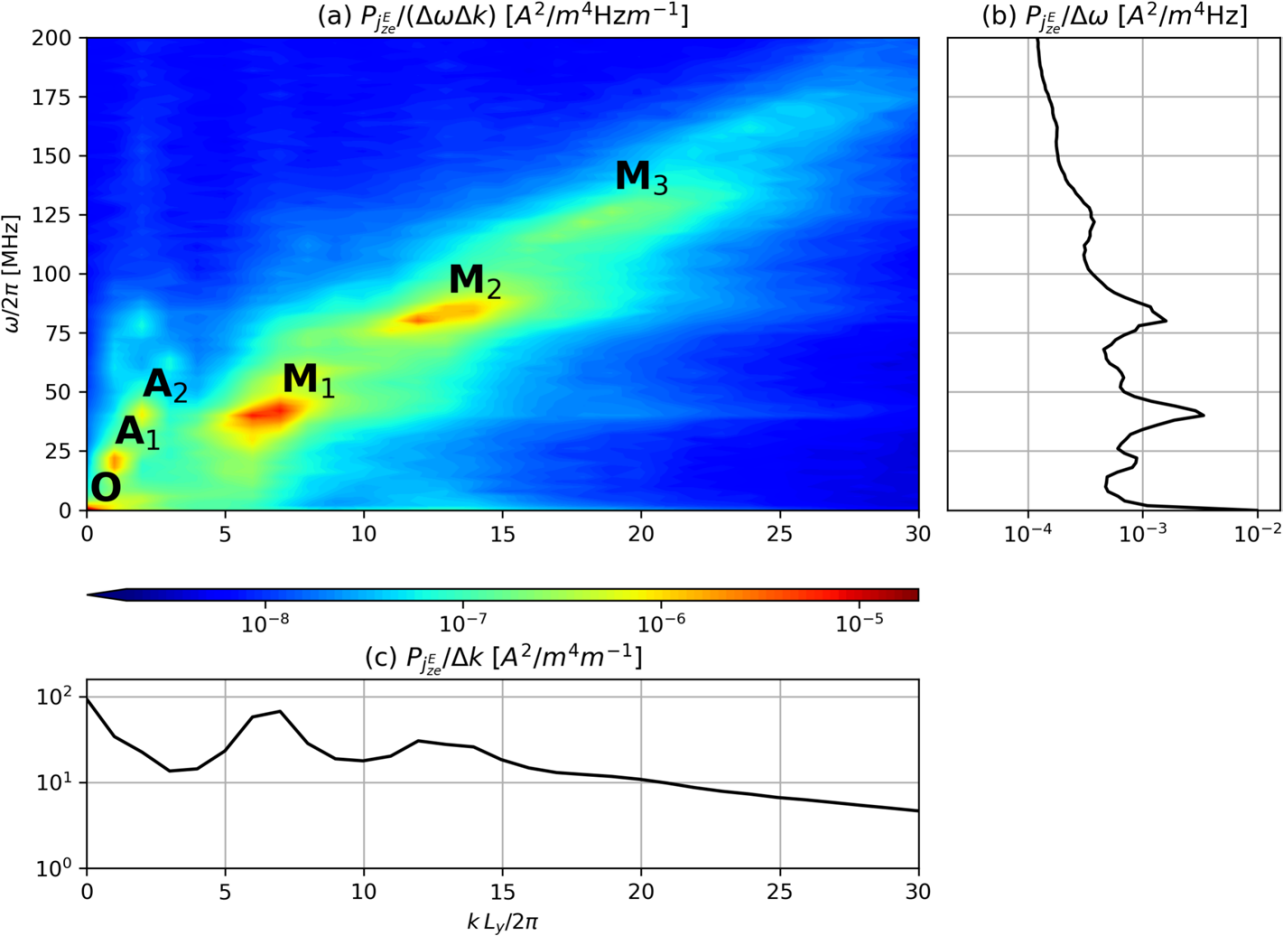
**a** Power spectral density (PSD) of $j_{ze}^{E}$ as taken from the axial slice at $z=1.98$ mm. Plots (**b**) and (**c**) showcase the resulting power from integrating over *k* and $\omega$, respectively. Modes *O*, $A_1$, $A_2$, $M_1$, $M_2$, $M_3$ correspond to the peaks identified in Fig. 2

In Fig. 4, the statistically estimated PSD of $j^{E}_{ze}$, $P_{j^{E}_{ze}}$, reveals that similar modes are present as in $\phi$ in Fig. 2b, albeit with varying amplitude. The auxiliary panels show the reduced PSD as a function of $\omega$ only or *k* only, which make it easier to appreciate that the DC component of $j^{E}_{ze}$ dominates the spectrum, over the other peaks which are associated, in order of importance, to modes $M_1$, $M_2$, and $A_1$. Because the frequency resolution is $\Delta \omega / (2\pi ) = 2$ MHz, the spectral power of mode *O* is mixed with the mean-flow component and cannot be easily distinguished in Fig. 2b.

As $j_{ze}^{E}$ is the product of $n_e$ and $E_{y1}$ save for a constant factor, its spectrum can be computed as the convolution of those of $n_e$ and $E_{y1}$,

20 $$\begin{aligned} \hat{\tilde{\jmath }}^{E}_{ze}(\omega _m, k_{n})=\frac{e}{B_0}\sum \limits _{l}\sum \limits _{p}\hat{\tilde{n}}_e(\omega _l, k_p)\hat{\tilde{E}}_y(\omega _m-\omega _l, k_{n}-k_p). \end{aligned}$$

Multiplying both sides by $\hat{\tilde{\jmath }}^{E *}_{ze}(\omega _m, k_{n})$ and averaging over multiple realizations we find the relation between $P_{j_{ze}^{E}}$ and the (four-dimensional) cross-bispectrum $\mathfrak {B}_{n_eE_yj^{E}_{ze}}$,

21 $$\begin{aligned} P_{j_{ze}^{E}}(\omega _m, k_{n})=\frac{e}{B_0}\sum \limits _{l}\sum \limits _{p} \mathfrak {B}_{n_eE_yj^{E}_{ze}}(\omega _l, \omega _m - \omega _l, k_p, k_{n} - k_p). \end{aligned}$$

**Fig. 5:**
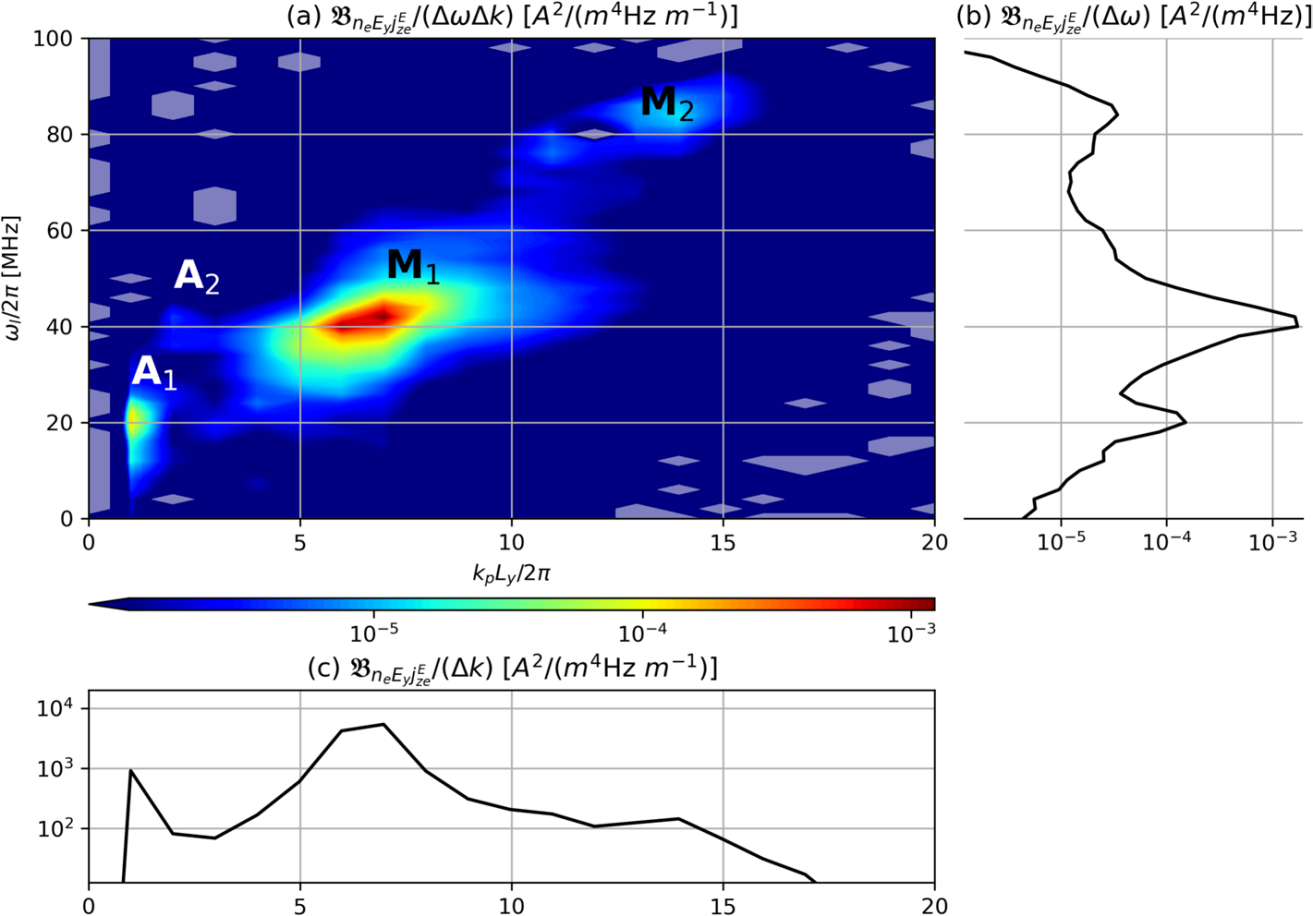
Cross-bispectrum $\mathfrak {B}_{n_eE_yj^{E}_{ze}}(\omega _l, - \omega _l, k_p, - k_p)$ of $n_e$, $E_{y}$ and $j_{ze}^{E}$, reflecting the spectral contribution to the mean-flow of the cross-field current. By construction, this slice of $\mathfrak {B}_{n_eE_yj^{E}_{ze}}$ is real. Areas shaded in white denote negative values. Plots (**b**) and (**c**) showcase the resulting power from integrating over $k_p$ and $\omega _l$, respectively. Modes $A_1$, $A_2$, $M_1$, $M_2$ correspond to the peaks identified in Fig. 2

This equation makes it manifest that the power in $P_{j_{ze}^{E}}$ for a particular mode $(\omega _m, k_{n})$ stems from all the quadratic phase coupling between $n_e$ and $E_y$ contained in a slice of the cross-bispectrum $\mathfrak {B}_{n_eE_yj^{E}_{ze}}$. In particular, Fig. 5 displays the contributions of each ($\omega _l$, $k_p$) in Eq. (21) to the mean-flow of $j_{ze}^E$, i.e., for $\omega _m=0$, $k_n=0$, with each point corresponding to the coupling of a mode of the electron density $n_e$ at $(\omega _l,k_p)$ and another of $E_y$ at $(-\omega _l, -k_p)$. Observe that all $\mathfrak {B}_{n_eE_yj^{E}_{ze}}$ contributions to the mean-flow are real.

These results allow us to conclude that, while linear ECDI theory predicts the dominance of $M_2$ due to a larger growth rate, the $M_1$ ECDI mode clearly dominates in the saturated state, and that the phase correlations between $n_e$ and $E_y$ in this mode are the leading contribution to the mean-flow anomalous transport. This peak contribution is followed by $A_1$ (one order of magnitude smaller) and $M_2$ (roughly two orders smaller). Observe that the near-axial mode *O* does not have any relevant contribution to anomalous transport. Similarly, the range above 100 MHz (not shown in the figure) does not seem to feature any relevant quadratic phase coupling.

### Three-wave power coupling

We now focus on elucidating the nonlinear interactions among the fluctuation modes in saturation that can explain the preponderance of $M_1$ in the spectrum and its contribution to anomalous transport, from a weak-turbulence framework [27]. This is relevant for understanding which are the actual sources of spectral energy in the nonlinear regime (i.e., instabilities) and the channels through which this energy flows from mode to mode, ending up in $M_1$, which as we have seen in Anomalous axial transport section, dominates anomalous transport.

**Fig. 6:**
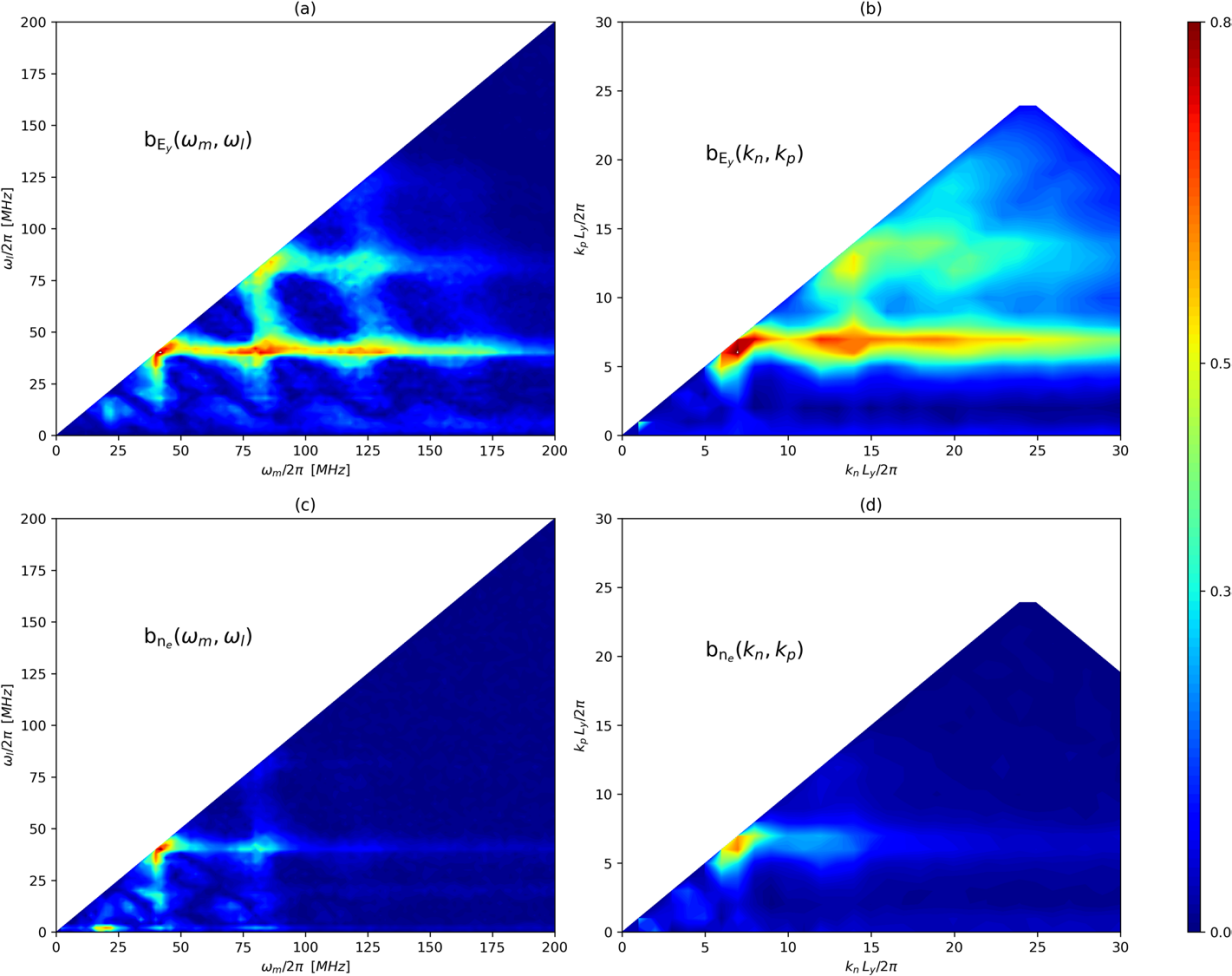
Reduced self-bicoherence of $E_{y}$ (**a**-**b**) and $n_e$ (**c**-**d**). Plots (**a**) and (**c**) are in $\omega$ while (**b**) and (**d**) plots in *k*. Only the regions of non-redundant information within the Nyquist limit are shown, see Spectral and bispectral analysis

Figure 6a-b showcases the reduced self-bicoherence of $E_y$ in the $\omega$ and *k* domains, in order to identify the quadratic couplings responsible for nonlinear power transfers that shape the saturated spectrum. The plots in $\omega$ and in *k* present roughly the same structure; the underlying reason is that the peaks in the (four-dimensional) bicoherence $b(\omega _m,\omega _l,k_n,k_p)$ fall along a subspace given by $\omega _m \propto k_n$, $\omega _l\propto k_p$, with a constant of proportionality equal to the phase velocity of the *M* modes. This permits us to refer equivalently to the (reduced) bicoherence properties in $\omega$ or *k*.

The structure of the bicoherence diagrams reveals the strong nonlinear interaction among modes $M_1$, $M_2$, $M_3$ (and even higher modes in the *M* branch). Specifically, mode $M_1$ has a coherent interaction with higher modes, as evidenced by horizontal strip at $\omega _l/(2\pi )=42$ MHz (respectively, $k_pL_y/(2\pi )=7$) with large bicoherence. We note that the resonant interaction between $M_1$, $M_2$ and $M_3$ must be understood in an broad sense, because the resonance conditions (4) are not strictly satisfied among the central $(\omega ,k)$ values of each mode, but only approximately. Indeed, this could be a cause of the observed broadening that the modes exhibit, and may suggest that the interaction involves various neighboring frequencies and wavenumbers, with a power transfer that undergoes various complex three-wave-coupling exchanges among them. Significantly weaker interactions are seen involving the *A*-branch modes, although relatively small peaks are present corresponding to the triad $\left( {A_1A_1} \rightleftharpoons {A_2}\right)$ and between $A_1$ and $A_2$ to their sum frequency, 60 MHz. Overall, the high values of the bicoherence (up to $b=0.8$) give us confidence in that three-wave coupling is a main feature of the late, nonlinearly saturated behavior of the discharge.

However, while a large bicoherence is a clear indicator of nonlinear power transfer among modes, an inherent limitation of this spectral quantity is that it does not discriminate by itself the direction in which the energy flows. To assess this particular aspect, it is necessary to reconstruct a (simplified) model of the spectral energy flow that features the corresponding three-wave coupling terms. The basic form of the three-wave coupling equations for the complex wave amplitudes [27] $\tilde{x}_i(z,t)$ in a $[z,z+\delta z]$ slab of the plasma, for three modes that feature a single resonance $({1,2} \rightleftharpoons {3})$ (conditions (4)), can be written as:

22 $$\begin{aligned} \frac{\partial }{\partial t}{\tilde{x}}_1 & =(\gamma _1 - \text {i} \omega _1) {{\tilde{x}}_1}-v_{gz1}{\frac{\partial }{\partial z}{\tilde{x}}_1}+ V_{1,23}{{\tilde{x}}_2{\tilde{x}}_3},\nonumber \\ \frac{\partial }{\partial t}{\tilde{x}}_2 & =(\gamma _2 - \text {i} \omega _2) {{\tilde{x}}_2}-v_{gz2}{\frac{\partial }{\partial z}{\tilde{x}}_2}+ V_{2,31}{{\tilde{x}}_3{\tilde{x}}_1},\nonumber \\ \frac{\partial }{\partial t}{\tilde{x}}_3 & =(\gamma _3 - \text {i} \omega _3) {{\tilde{x}}_3}-v_{gz3}{\frac{\partial }{\partial z}{\tilde{x}}_3}+ V_{3,12}{{\tilde{x}}_1{\tilde{x}}_2}. \end{aligned}$$

The coefficients on the right-hand sides are the linear growth rate $\gamma _i$ and frequency $\omega _i$, the *z* component of the group velocity $v_{gzi}$ of each mode *i*, and the resonant wave-wave interactions with coupling coefficients $V_{i,jk}$ with the other two modes. Note that no advection of power in the *y* direction appears explicitly in this expression, as we are modeling a plasma slab at a *z* location. Also observe that naturally, in an actual plasma, there can be more than one quadratic coupling affecting each mode, and there can exist higher-order nonlinear interactions (e.g. cubic, quartic) so a complete model should account for additional interactions. Nevertheless, in this work we seek a simplified model of the three dominant *M* modes based on expressions (22), which allow for two possible quadratic interactions: $\left( {M_1M_1} \rightleftharpoons {M_2}\right)$ and $\left( {M_1M_2} \rightleftharpoons {M_3}\right)$. The corresponding two terms are taken into account in the following.

Multiplying the first of the Eqs. (22) by $\tilde{x}_1^*$ after including the two possible resonances that affect mode 1 gives, after some manipulation:

23 $$\begin{aligned} \frac{\partial }{\partial t}|\tilde{x}_1|^2=2\gamma _1 |\tilde{x}_1|^2-v_{gz1}\frac{\partial }{\partial z}|\tilde{x}_1|^2 + 2V_{1,12} \cos \alpha _{112} |\tilde{x}_1 \tilde{x}_1 \tilde{x}_2| + 2V_{1,23} \cos \alpha _{123} |\tilde{x}_1 \tilde{x}_2 \tilde{x}_3|, \end{aligned}$$

(and similarly for the remaining two equations), where $\alpha _{112}$ and $\alpha _{123}$ are the mutual phase angle resulting from the phases of the modes. Our goal is to determine the value of the parameters appearing in this equation from the simulation data. However, determining these coefficients by standard data regression methods faces two main challenges: firstly, the algebraic system represented by expression (22) is typically overdetermined when considering multiple time instants and/or realizations. Secondly, for strictly stationary data, the terms $\partial /\partial t$ become negligible compared to the terms on the right-hand side, making the problem ill-conditioned (i.e., if the left hand side for all equations were exactly zero, coefficients can be determined up to a multiplying factor only). This latter issue is not usually acknowledged in the literature of nonlinear interactions when reconstructing the coefficients from data. The prevailing regression schemes are due to Kim and Ritz [37, 38] and De Wit [39]. In essence, they obtain a single fit for the $\gamma _i$, $\omega _i$, $v_{gzi}$ vectors and the $V_{i,jk}$ matrix simultaneously from several higher-order spectra for all possible modes in the problem; as a consequence, these methods are usually computationally expensive. Furthermore, they typically result in full matrices with an enormous list of terms on each mode’s equation, which complicates their interpretability: these methods do not discriminate which terms in each mode’s equation are more fundamental and which are accessory (other than looking at the the magnitude of the coefficients themselves). That is, we have no clear indication of how the fit error would deteriorate, were we to drop one particular term in the equation. This question is relevant when we intend to derive simple, understandable models that capture the dominant nonlinear dynamics with the smallest possible number of terms.

Here instead we use the SINDy algorithm and Pareto front analysis to identify a hierarchy of reduced models for the power spectral density $P_{E_y}(t,k)$ of the electric field $E_y$ of the dominant $M_1$, $M_2$ and $M_3$ modes only at the slab located at $z=1.98$ mm. While this does not remove the ill-posedness of the regression discussed above, it allows us to effectively and efficiently order the right hand side terms according to their significance in the dynamics, and to truncate the hierarchy at a sensible point that balances model accuracy and complexity. To take mode broadening into account, we select a *k* interval of $\Delta kL_y/2\pi =1$ around each mode $M_1$, $M_2$, $M_3$ and average $P_{E_y}(t,k)$ over them to obtain $P_i(t)$ ($i=1,2,3$). These $P_i$ are finally averaged on small time windows spanning 1.4 $\mu$s with 80% overlap to obtain a time signal that can be used in the SINDy algorithm. The averaging and the time windowing has the additional advantage of reducing the susceptibility of the results to noise, as in the “weak-SINDy” variant of the technique [40]. Sensitivity analysis within a $\pm 0.1\mu s$ of the time averaging parameters confirmed the stability of this scheme and of the resulting coefficients.

Reasoning from Eq. (23), we take the ansatz that the mode interactions remain moderately coherent over time [27] such that $\cos \alpha _{112}$ and $\cos \alpha _{123}$ in equation (23) remain essentially constant. This assumption is supported by the large bicoherence values found among the modes, which would diminish if the mutual phase were not stable. With this premise, with $P_i(t)$ taking the place of $|\tilde{x}_i|^2$ in Eq. (23), we can expect $P_1$ to follow an evolution law analogous to

24 $$\begin{aligned} \frac{\partial }{\partial t}P_1 \approx 2 \gamma _1 {P_1}-v_{gz1}{\frac{\partial }{\partial z}P_1}+2V_{1,12}^{\prime } {\sqrt{P_1P_1P_2}} + 2V_{1,23}^{\prime } {\sqrt{P_1P_2P_3}} \end{aligned}$$

(and similar expressions for modes $M_2$ and $M_3$).

To apply the SINDy algorithm to separately identify the evolution equations for the $P_i$ of each mode, we create a feature library including all possible linear and quadratic combinations of the $P_i$, as well as the axial derivatives $\partial P_i/\partial z$. The derivative terms of $P_i$ are computed using second-order centered finite differences applied to neighboring axial positions and time snapshots.

The best models found for $M_1$, $M_2$, and $M_3$ with up to 4 terms on the right hand side are shown in Table 2, together with their regression error $\varepsilon ^{S}_i$. As discussed in the Sparse Identification of Nonlinear Dynamics (SINDy) section, this constitutes a Pareto front (or hierarchy) of models of increasing complexity and decreasing error. We have highlighted in bold our identified Pareto knee model for each mode, which corresponds to the biggest decrease in error by the addition of a single term. The relative importance of a term in its respective equation (i.e. its relevance in the fit) is represented by how early it appears in the hierarchy. Note that once $\varepsilon ^{S}_i$ has saturated (that is, when it does not decrease significantly with the addition of new terms), experience dictates a loss of physical meaningfulness with additional terms, and usually results from overfitting the data.

**Table 2:**
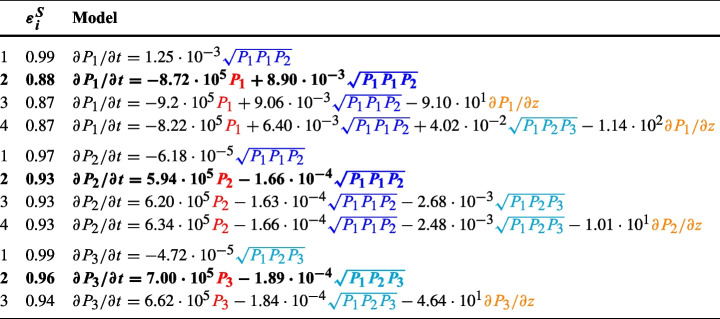
Pareto front models for modes $M_1$, $M_{2}$, $M_{3}$, obtained by sparse regression

$P_i$ denotes the power spectral density of mode *i*. Terms in are associated to linear growth/decay rates;  designates convection terms;  and  terms highlight related wave-wave couplings terms across the modes. The Pareto-optimal models are highlighted in **bold**. The units of the coefficients are 1/s for $\gamma _i$, m/s for $v_{gzi}$ and m/Vs for $V_{i,jk}$, see Eq. 24.

The fact that $\varepsilon ^{S}_i$ decreases and then stabilizes beyond the Pareto knee model suggests reliable model identification [32, 33] and therefore the capture of the dominant power dynamics of the *M* modes. However, the relatively large error of the resulting models (0.88–0.96) indicates that linear growth/decay terms, three-wave interactions among these three modes only, and axial advection do not provide a complete description of the power dynamics of the *M* branch. Including higher-order coupling terms and interactions with other modes beyond these three are likely needed to decrease the error further. Nevertheless, our attempts to include the interaction terms with the *A* modes and the *O* mode did not yield satisfactory models, likely because the coupling with these terms is small as evidenced by the bicoherence diagrams in Fig. 6a. Likewise, a finer-grained characterization of the spectrum (e.g. splitting each broadband *M* mode into its various ($\omega ,k$) would yield a better agreement with the data at the cost of interpretability.

Given the ill-posedness of the regression problem and the large regression error $\varepsilon ^{S}_i$, we refrain from making quantitative claims in favor of a more qualitative analysis of the resulting models. In particular, we focus on the order of appearance of the terms in the hierarchy, and the sign and magnitude of the coefficients, where positive/negative signs indicate energy flow into/out of the mode, respectively.

In the model equations for $M_1$ and $M_2$, the dominant term (and therefore the first that shows up in the hierarchy) is the coupling term for the triad $\left( {M_1M_1} \rightleftharpoons {M_2}\right)$. This coupling transfers energy from mode $M_2$ into mode $M_1$ according to the sign structure of the coefficients. The term coupling modes $\left( {M_1M_2} \rightleftharpoons {M_3}\right)$ appears first in the model for mode $M_3$, and also shows up for models more complex than the Pareto-optimal ones for $M_1$ and $M_2$. This term would convey energy from $M_3$ and $M_2$ into $M_1$.

The term that appears second in the hierarchies of the three modes is the linear term in $P_i$, which corresponds to the coefficient of the growth/decay rate $\gamma _i$, and its inclusion suffices to reach the prescribed Pareto optimality on all three models. The growth rate represents the balance between the power input from the instability to each mode, and the power loss to generate anomalous transport. The signs indicate *net* power input into modes $M_2$ and $M_3$ and dissipation in mode $M_1$.

The reminder of terms (i.e., those appearing for the first time in the last equations of the hierarchy of each model) come after Pareto optimality, and therefore may well represent model overfitting. Nevertheless, and with this warning in mind, we may dare a cautious look at them to find that axial convection across the slab, at least at this position ($z= 1.98$ mm), is deemed to play an overall minor role. The sign of these terms would suggest power advection in the negative *z*-direction, corresponding with the direction of the local *z* wave number in our data.

Regarding the magnitude of the terms, we note that if these three modes were an isolated system with no noise in the data, we would expect exact energy conservation, with the coefficients for each triad in each equation adding up to zero. This is clearly not so, as the coupling coefficients differ in at least one order of magnitude for each of the two nonlinear couplings recovered in the model. Apart from the error of the fit, to which part of this incongruence can be likely attributed, we stress that this reduced model does not cover for all the nonlinear power couplings likely affecting these three modes, and that a model displaying energy conservation would need to take into account all possible interactions, which is beyond the scope of our simple fitted model. On the other hand, the magnitudes of the (net) growth rates $\gamma _i$ are comparable among the three modes, and two orders of magnitude lower than predicted by linear theory (see Fig. 2d). This could be attributed by the fact that all three modes (and specially $M_1$) contribute to anomalous transport, and this brings in a loss of power in the modes. A similar decrease in the growth rate at saturation with respect to linear theory has been seen experimentally [13, 15].

**Fig. 7:**
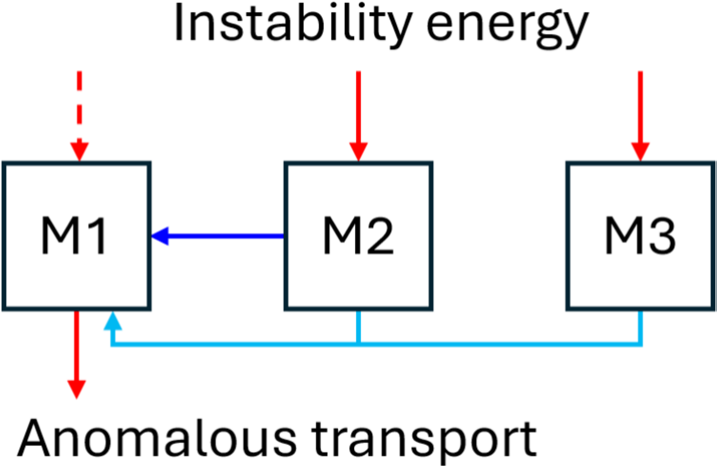
Schematic of the power flux in the Pareto-optimal reconstructed model in Table 2, with arrows representing energy transfer.

From the qualitative analysis of the linear growth rate and the quadratic coefficients, a general picture emerges: the sign structure of the couplings $\left( {M_1M_1} \rightleftharpoons {M_2}\right)$ and $\left( {M_1M_2} \rightleftharpoons {M_3}\right)$ suggest an inverse energy cascade, where energy of the higher-frequency, higher-*k* modes flows toward lower-frequency, lower-*k* modes. Modes $M_2$ and $M_3$ receive net energy from the ECDI instability, which is then transferred to the longer-scale mode $M_1$. The negative growth rate of mode $M_1$ can be attributed to a net energy loss between what it gains from the instability and what it expends to generate anomalous plasma transport. This energy flow has been schematically represented in Fig. 7.

One may wonder to what extent this model is representative of the dynamics of our $\varvec{E}\times \varvec{B}$ plasma, given the absence of the *A*-branch and mode *O* from the model. However, as advanced above, our attempts to include them in the models of the *M* brach yielded no satisfactory result (probably due to the small presence of the *A* and *O* modes in the bicoherence diagram, Fig. 6a).

Finally, we return to Fig. 6c-d to discuss the structure of the self-bicoherence of $n_e$. Compared to $E_y$, $n_e$ presents significant differences. In fact, while the triad $\left( {M_1M_1} \rightleftharpoons {M_2}\right)$ is still prominently present, the rest of the interactions among the *M*-branch display significantly lower bicoherence than in $E_y$. Moreover, there is a strong, localized interaction in this case among mode *O* and $A_1$ near the horizontal axis of $b_{n_e}(\omega _m,\omega _l)$, and also a weaker one between *O* and $M_1$, $M_2$. These differences among $E_y$ (field) and $n_e$ (plasma) hint at the possibility that a two-field description of the nonlinear dynamics may be necessary to fully explain the nonlinear interactions and energy fluxes among the modes in saturated ECDI scenarios like this one, and perhaps to fully elucidate the coupling among mode branches, which appear to be essentially independent in the analysis of $E_y$ alone.

## Summary

Our analysis of an $\varvec{E}\times \varvec{B}$ plasma kinetic simulation identified two distinct dispersion branches together with a low-frequency mode that modulates the discharge. Anomalous transport was seen to be primarily driven by in-phase fluctuations in density and electric field arising from the longest-wavelength mode of the Electron Cyclotron Drift Instability (ECDI) branch, $M_1$. Other modes like $A_1$ and $M_2$ contribute weakly. The axial electron current is seen to be modulated by mode *O*, and the *M* and *A* branches appear to alternate in time.

There is a strong bicoherence among *M* modes in the $E_y$ spectrum, and sparse regression (SINDy) was employed to derive a hierarchy of data-driven spectral models for the quadratic power transfer among them. We conclude that these wave-wave interactions are a key aspect of the mode dynamics, and that the most likely structure of this energy flow is an inverse energy cascade. Nevertheless, the high fit error suggest that other mechanisms are likely involved in the power transfer as well, such as higher-order interactions.

The capability of the bispectral analysis to identify and isolate the different mode contributions to anomalous transport can be of use for studies with co-existing instabilities, such as ECDI combined with lower hybrid or modified two stream instabilities [15, 17, 41]. Additionally, the SINDy approach for determining growth rates and nonlinear energy exchange directionality can be valuable for the understanding of general oscillatory dynamics. The methodology used here is therefore deemed directly applicable to other plasma transport studies driven by fluctuations. Future research could refine present results by incorporating wavelet analysis to better account for temporal variations of broadened structures.

## Data Availability

The data that support the findings of this study are openly available at the following URL/DOI: 10.5281/zenodo.14678231.
